# Fracture fixation strategy and specific muscle tissue availability of neutrophilic granulocytes following mono- and polytrauma: intramedullary nailing vs. external fixation of femoral fractures

**DOI:** 10.1186/s40001-020-00461-y

**Published:** 2020-11-26

**Authors:** Johannes Greven, Klemens Horst, Zhi Qiao, Felix Marius Bläsius, Ümit Mert, Michel Paul Johan Teuben, Nils Hendrik Becker, Roman Pfeifer, Hans-Christoph Pape, Frank Hildebrand

**Affiliations:** 1 Department of Trauma and Reconstructive Surgery, RWTH Aachen University Hospital, Pauwelstraße 30, 52074 Aachen, Germany; 2 Department of Orthopedics, First Affiliated Hospital of Zhengzhou University, Jianshe East Road, Zhengzhou City, 450052 China; 3 Department of Traumatology, University Hospital Zurich, Rämistrasse 100, 8091 Zurich, Switzerland

**Keywords:** Neutrophil granulocyte, Intra medullary nailing, External fixation, Trauma, Femoral fracture

## Abstract

**Background:**

In the stabilization of femoral fractures in mono- and polytrauma, clinical practice has shown better care through intramedullary nailing. However, the reason why this is the case is not fully understood. In addition to concomitant injuries, the immunological aspect is increasingly coming to the fore. Neutrophil granulocytes (PMNL), in particular next to other immunological cell types, seem to be associated with the fracture healing processes. For this reason, the early phase after fracture (up to 72 h after trauma) near the fracture zone in muscle tissue was investigated in a pig model.

**Material and methods:**

A mono- and polytrauma pig model (sole femur fracture or blunt thoracic trauma, hemorrhagic shock, liver laceration, and femur fracture) was used to demonstrate the immunological situation through muscle biopsies and their analysis by histology and qRT-PCR during a 72 h follow-up phase. Two stabilization methods were used (intramedullary nail vs. external fixator) and compared with a nontraumatized sham group.

**Results:**

Monotrauma shows higher PMNL numbers in muscle tissue compared with polytrauma (15.52 ± 5.39 mono vs. 8.23 ± 3.36 poly; *p* = 0.013), regardless of the treatment strategy. In contrast, polytrauma shows a longer lasting invasion of PMNL (24 h vs. 72 h). At 24 h in the case of monotrauma, the fracture treated with external fixation shows more PMNL than the fracture treated with intramedullary nailing (*p* = 0.026). This difference cannot be determined in polytrauma probably caused by a generalized immune response. Both monotrauma and polytrauma show a delayed PMNL increase in the muscle tissue of the uninjured side. The use of intramedullary nailing in monotrauma resulted in a significant increase in IL-6 (2 h after trauma) and IL-8 (24 and 48 h after trauma) transcription.

**Conclusion:**

The reduction of PMNL invasion into the nearby muscle tissue of a monotrauma femur fracture stabilized by intramedullary nailing supports the advantages found in everyday clinical practice and therefore underlines the usage of nailing. For the polytrauma situation, the fixation seems to play a minor role, possibly due to a generalized immune reaction.

## Background

Delayed fracture healing represents a frequent complication after high-energy trauma [1, 2]. In addition to patient-related (e.g., diabetes, smoking) and fracture-related (e.g., open fractures) factors, concomitant injuries (e.g., chest trauma) and stabilization strategies (e.g. intramedullary stabilization, and external fixation) may also affect fracture healing. Among the potential pathophysiological processes, the local immunological response has been particularly suspected to be of major relevance. In the early pro-inflammatory phase of fracture healing, neutrophil granulocytes (PMNL) are one of the first immune cells recruited into the fracture site. There, they form an extracellular "emergency matrix" by releasing fibronectin, which provides a structure even before the migration of connective tissue cells [3]. The initiation of this mobilization and migration of PMNL depends on interleukin (IL)-8 [4, 5].

Various studies have shown that the number and activity of the PMNL at the fracture site are important in the fracture healing process [3, 5–9]. In this context, the reduced number of PMNL in the fracture hematoma has been associated with insufficient callus formation due to a disturbed conversion from connective tissue into bony substances [9]. However, excessive PMNL numbers in the fracture hematoma also negatively influence fracture healing, most likely due to an increased release of reactive oxygen species (ROS) associated with enhanced damage of the surrounding tissue. These processes might even be further aggravated by a longer survival time of PMNL due to a generally increased post-traumatic expression of antiapoptotic genes [10].

A good coverage of the bone with muscle tissue is particularly relevant for fracture healing. The musculature supplies the bony tissue with oxygen and nutrients as well as with osteoprogenitor cells, but the humeral (e.g., IL-6-secretion) and cellular immunological processes ongoing in the muscle tissue also seem to be fundamental in fracture healing [11]. Among the cellular components, the infiltration of PMNL into the musculature seems to be critical for the induction of regenerative processes, such as fracture healing [9]. However, enhanced post-traumatic PMNL migration also has the potential to damage muscle tissues. Therefore, the modulation of PMNL migration might play an essential role in ensuring the balance between excessive inflammation and healing [4].

Nevertheless, for fracture hematoma it was shown that it might have different cytokine patterns then other fracture surrounding tissues and thus also different cellular compositions than those in the muscle tissue [12]. Consequently, in addition to the musculature, the fracture hematoma and maybe additional soft tissues are also of particular importance for trauma outcome.

Despite the known relevance of the musculature, the kinetics of muscular PMNL infiltration at the fracture site remains unclear. Similarly, the specific impact of concomitant injuries, as well as effects of the choice of surgical strategy for fracture fixation, on muscular PMNL concentrations are not known. We therefore investigated these aspects in a unique and clinically relevant large animal model following induction of either an isolated femoral monotrauma fracture or a polytrauma.

## Materials and methods

### Animal care

All experiments were performed in accordance with the German legislation governing animal studies, following the “Principles of Laboratory Animal Care” [13]. Official permission was granted by the North Rhine-Westphalia State Office for Nature, the Environment and Consumer Protection (Landesamt für Natur, Umwelt und Verbraucherschutz Nordrhein-Westfalen, Recklinghausen, Germany, project number: 84.02.04.2014 A265), which also approved all experimental protocols. Male German landrace pigs (German Landrace *Sus scrofa*) with a body weight of 30 ± 5 kg were housed with a 12 h day/night rhythm 7 days before the experiments to allow acclimatization to their surroundings. Pre-infection was excluded by a veterinarian examination of all animals before the experiments started. The data presented in this paper were collected in the context of a larger study [14] for the benefit of the principles of the 3Rs (Replacement, Refinement, and Reduction) [15].

### General instrumentation, anesthesia, and surgical procedures

The experimental setup was established and validated at the Department of Trauma and Reconstructive Surgery, RWTH, Aachen and was described in detail by Horst et al. in 2016 [14]. Prior to the experiment, animals were premedicated with an intramuscular injection of azaperone. Anesthesia then was induced by an intravenous injection of propofol followed by orotracheal intubation (7.5 ch; Hi-Lo LanzTM). Vital parameters were monitored by electrocardiographic (ECG) recordings and ECG-synchronized pulse oximetry, as previously described [14]. A central venous line was placed into the right jugular vein (Four-Lumen Catheter, 8.5 Fr., Arrow Catheter, Teleflex Medical, Germany). A three-lumen hemodialysis catheter (12.0 Fr., Arrow Catheter, Teleflex Medical, Germany) was also placed in the right femoral vein to induce hemorrhage, and an arterial line (Vygon, Aachen, Germany) was placed in the femoral artery for continuous monitoring of blood pressure. A suprapubic bladder catheter was also placed. Anesthesia was maintained with propofol and sufentanil during the entire study period. Fluids were administered by continuous crystalloid infusion (Sterofundin ISO®).

### Experimental groups

Pigs either sustained monotrauma (isolated femur fracture, *n* = 12) or polytrauma (femoral fracture, chest and abdominal trauma, hemorrhage of max. 45% of the total blood volume, *n* = 12). Noninjured animals that received the same instrumentation, medication, and treatment, but no trauma, served as sham group (*n* = 6). The fractures sustained by the “Monotrauma” and “Polytrauma” groups were stabilized either by external fixation (Radiolucent Fixator, Orthofix, *n* = 6) or by intramedullary nailing (T2 System, Stryker, *n* = 6). In addition to the sham group, four experimental groups were included: Monotrauma Nailing (Mono_N, *n* = 6), Monotrauma External Fixation (Mono_Ex_fix, *n* = 6), Polytrauma Nailing (Poly_N, *n* = 6), and Polytrauma External Fixation (Poly_Ex_fix, *n* = 6).

### Trauma induction and 72 h ICU phase

After achieving stable baseline values (at least 120 min after instrumentation) for O_2_ at 21% during the trauma period, simulating the ambient air, either monotrauma or multiple trauma was induced. The femur fracture was achieved using a bolt gun machine (Blitz-Kerner, turbocut JOBB GmbH, Germany) and cattle-killing cartridges (9 × 17; DynamitNobel AG, Troisdorf, Germany). The bolt hit a custom-made punch positioned on the mid third of the femur [14], for polytrauma, a pair of panels (steel: 0.8 cm and lead: 1.0 cm thickness) was placed on the right dorsal, lower chest. A bolt was shot onto this panel, simulating a blunt lung contusion. An additional laparotomy was performed to approach the liver and the mid-lobe of the liver was cut crosswise (4.5 × 4.5 cm) to half of the liver thickness in depth, with uncontrolled bleeding allowed for 30 s. The liver was then packed with 10 × 10 cm gauze and the laparotomy was closed. A pressure-controlled and volume-limited hemorrhagic shock was then induced by withdrawing blood until a mean arterial pressure (MAP) of 40 ± 5 mmHg was reached. In this context, a maximum of 45% of the total blood volume was drawn from the left femoral vein. The shed blood was kept in blood bags for reinfusion purposes. Hemorrhagic shock was maintained for 90 min [14].

After this period, the animals were resuscitated in accordance with established trauma guidelines (ATLS® & AWMF-S3 guideline on Treatment of Patients with Severe and Multiple Injuries®) [16]. The animals were rewarmed using a forced-air warming system until normothermia (38.7–39.8 °C) was reached [16]. In addition to crystalloids (SterofundinISO and pediatric electrolyte solution 2 ml/kg BW/h), the pigs received previously withdrawn blood to restore hemostasis. The animals were mechanically ventilated and monitored in a special intensive care unit (ICU) for 72 h post-injury according to well-established ICU treatment guidelines. Antibiotics (Ceftriaxon® 2 g, i.v.) were administered before surgery and then every 24 h until the end of the experiment.

### Sampling

Muscle samples from the vastus lateralis muscle were taken clockwise at equal intervals in the area of the femoral fracture (traumatic [T]-side). Muscle samples were also taken from the contralateral femur from identical parts of the lateral vastus muscle (atraumatic [AT] side). Muscle samples of approximately 1 cm × 0.5 cm fixed in 4% formaldehyde for 24 h before embedding into paraffin blocks.

### Naphthol A-SD-chloroacetate esterase staining (CAE) histology

Sections (5–10 µm thick) of the muscle preparations were histologically stained with CAE stains to visualize PMNL that had migrated into the tissue. The paraffin-embedded tissue was deparaffinized using xylene (8 min), 100% ethanol (EtOH; 3 × 3 min), 95% EtOH (2 × 3 min), 70% EtOH (2 × 3 min), and phosphate buffered saline (PBS; 1 × 5 min). Solution 1 contained 5 ml PBS, 12.5 µl 4% sodium nitrite, and 12.5 µl New Fuchsin. Solution 2 contained 225 µl naphthol-AS-D chloroacetate in 5 ml PBS. The final stain contained 25 µl of solution 1 and 5 ml of solution 2.

The excess PBS on the slides with the samples was allowed to dry and the chloroacetate solution was added dropwise and the slides were incubated at room temperature (RT) for 45 min.

As a contrast stain, the slides were washed in PBS for 3 min and stained with Harris hematoxylin solution for 30–60 s and then immersed in 5 × saturated lithium carbonate solution, followed by washing with distilled water. The slides were dehydrated in 70% EtOH, 95% EtOH, 100% EtOH, and xylene for 10 short washing steps each and then covered with Permount mounting medium.

### PMNL scoring of muscle tissue

The average value of the cells identified as PMNL in a field of view (0.196 mm^2^) at 400 × magnification was chosen for scoring. Here, the important factors were to differentiate between the signal strengths of the different cell types and to count only strongly stained cells as PMNL. The PMNL count was averaged over 6 fields of view for each sample. This classification is also called “neutrophils per field of view” or the high-power field (N/HPF) score. According to the guidelines of the Musculoskeletal Infection Society (MSIS), scorings of more than 5 N/HPF are considered to represent a strong inflammatory reaction [142]. Scoring was performed by investigators JG and ZQ.

### Evaluation of the “monotrauma” groups by qRT-PCR

Concomitant injuries are known to affect cytokine transcription at the fracture site [17, 18]; therefore, our aim was to independently evaluate the influence of the fracture fixation strategy (nailing vs. external fixation) on the muscular transcription level of pro-inflammatory cytokines (IL-6 and IL-8). Therefore, qRT-PCR was performed only for muscle tissues subjected to monotrauma (Table 1).

**Table 1:** PCR primers (in the 5′ – 3′ direction)

Primer	Sequence 5′-3′
*Sus scrofa domesticus*	
β-Actin forward	GGACTTCGAGCAGGAGATGG
β-Actin reverse	GCACCGTGTTGGCGTAGAGG
RSP 18 forward	GGGTGTAGGACGGAGATAT
RSP 18 reverse	ATTACACGTTCCACCTCATC
HPRT forward	GTCAAGCAGCATAATCCAAAG
HPRT reverse	AAGGGCATAGCCTACCACAA
PPIA forward	AGCACTGGGGAGAAAGGATT
PPIA reverse	AAAACTGGGAACCGTTTG
Interleukin 6 forward	GAATCCAGACAAAGCCACCA
Interleukin 6 reverse	GTGCCCCAGCTACATTATCC
Interleukin 8 forward	ACTGCTGTTGTTGTTGCTTC
Interleukin 8 reverse	ATATCTGTACAACCTTCTGC

The PCR was run with the specified primers on a StepOne Plus RT device

### Statistics testing

First the obtained results were tested for normal distribution using the Kolmogorov–Smirnov test. Based on the not normally distributed values and the group size the Wilcoxon–Mann–Whitney test method was used for further calculation of the significance. The statistical significance was set at an error probability of *p* = 0.05 or *α* = 5%.

The calculated data were analyzed using SPSS and Microsoft Excel.

## Results

**Fig. 1:**
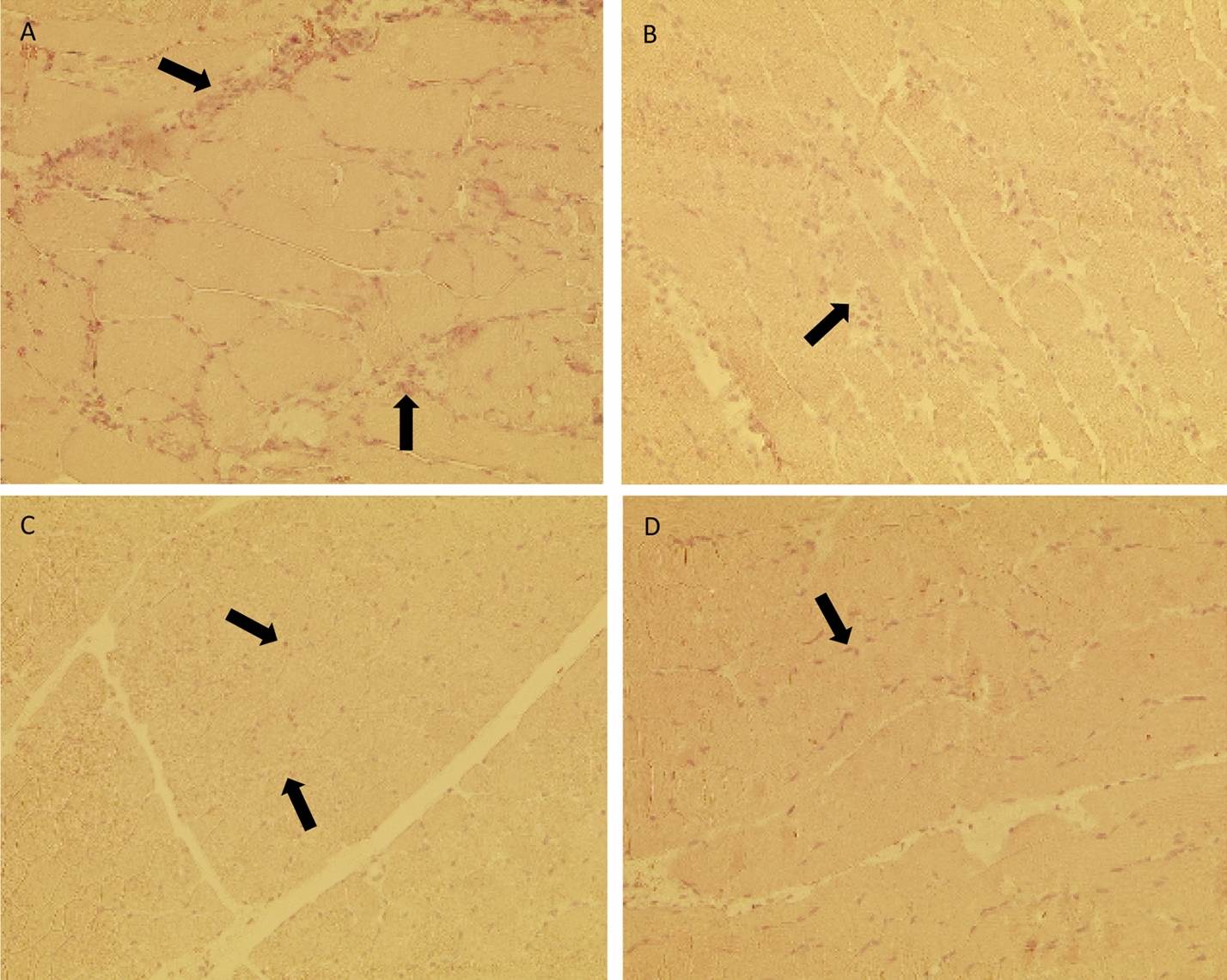
Histologic transmitted light image of CAE stained muscle tissues close to the fracture. **a** Muscle tissue preparation of the vastus lateralis muscle located close to the fracture of the Mono_Ex_fix is shown. **b** CAE staining of the muscle tissue of the Mono_N. **c** CAE staining of the muscle tissue of the Poly_Ex_fix. **d** CAE staining of the muscle tissue of the Poly_N. Cells marked with arrows are segment-nucleated and rod-shaped-nucleated neutrophils, which appear reddish due to the CAE staining (magnification 400 ×)

The histology was evaluated using 192 counting fields of 60 thin sections stained with CAE (Fig. 1).

### CAE staining and histology of muscle samples

### Absolute PMNL numbers in histological muscle samples

**Fig. 2:**
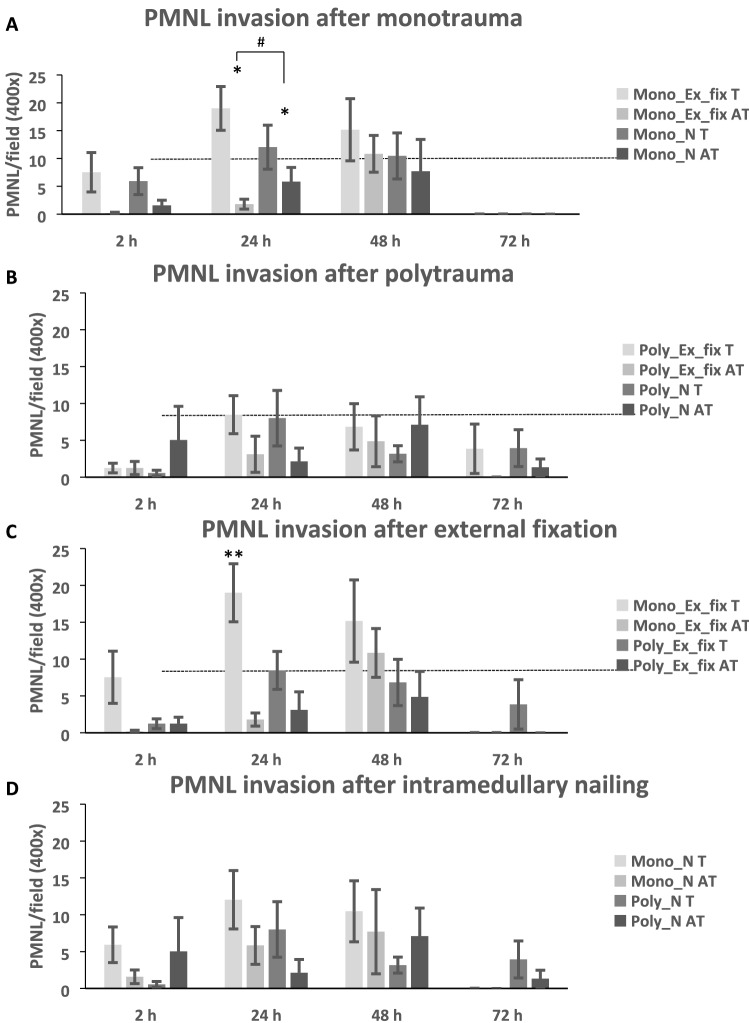
Neutrophil invasion in the musculus vastus lateralis after trauma. **a** Neutrophil invasion in muscle tissue after monotrauma for different fixation strategies (Mono_N; Mono_Ex_fix) and injury (T = trauma; AT = nontraumatized) at 2, 24, 48, and 72 h post-trauma. **b** Neutrophil invasion into muscle tissue close to the fracture in polytrauma with intramedullary nailing (Poly_N) and external fixation (Poly_Ex_fix) during the 72 h post-trauma observation period. **c** PMNL invasion after mono- and polytrauma after external fixator treatment of the femur fracture. **d** PMNL invasion of mono- and polytrauma after internal fixation. All values above the dotted line (> 5 N/HPF) indicate severe inflammation (*(**a**) Mono_Ex_fix 24 h T vs. AT *p* = 0.004; Mono_N 24 h T vs. AT *p* = 0.017, Mono_N T vs. Mono_Ex_fix T (*p* = 0.004). **24 h post-trauma: Mono_Ex_fix vs. Mono_N (*p* = 0.026), Poly_Ex_fix (*p* = 0.002), Poly_N (*p* = 0.015))

The histological PMNL counts in the different groups are presented in Fig. 2. The sham animals showed counts between zero and one PMNL per field (400 × magnification) in all evaluated sections.

In general, on the T side, all groups showed a maximum PMNL migration into the muscle tissue after 24 h (Fig. 2), with a subsequent steady decrease until 72 h after trauma. On the AT side, the neutrophil count showed a delayed increase, with a maximum at 48 h and a subsequent reduction until the end of the observation period. These different courses resulted in a significant difference in PMNL count at 24 h between the T and AT side for both monotrauma groups (Mono_Ex_fix 24 h T vs. AT *p* = 0.004; Mono_N 24 h T vs. AT *p* = 0.017).

Especially at 24 h, but also at 48 h, statistically significant higher neutrophil counts were observed for monotrauma than for polytrauma (pooled 15.52 ± 5.39 mono vs. 8.23 ± 3.36 poly; *p* = 0.013).

The greatest PMNL infiltration was found for the Mono_Ex_fix group, which showed a significant difference compared with Mono_N (*p* = 0.026), as well as to both polytrauma groups at 24 h (Poly_Ex_fix [*p* = 0.002], Poly_N [*p* = 0.015]).

In contrast to the monotrauma conditions, the fracture fixation strategy did not additionally affect PMNL migration in the polytrauma groups, either on the T side or on the AT side at 24 h. With the exception of the AT side after external fixation, a more prolonged infiltration of neutrophils occurred for polytrauma than for monotrauma, with PMNL counts still detectable at 72 h in the polytrauma groups.

**Fig. 3:**
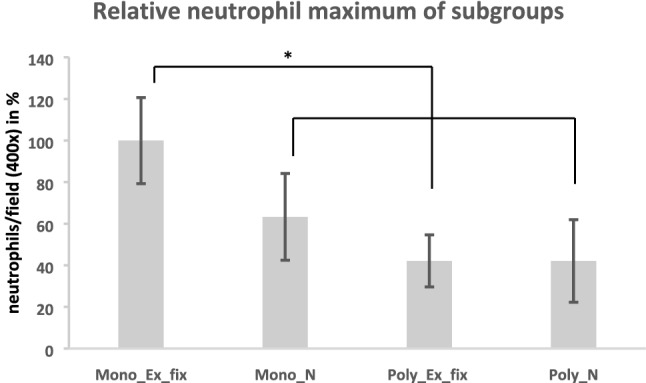
Maximum neutrophil number of the subgroups. Relative number of neutrophil granulocytes after 24 h in the muscle tissue close to the fracture of the different subgroups (mono/polytrauma; T/AT) in direct comparison. (*p < 0.05) (100% monotrauma ex. Fix.) (neutrophil granulocytes were counted at 400 × magnification)

The 5 N/HPF standard, established by the Musculoskeletal Infection Society (MSIS) [8], was also exceeded more clearly and frequently in the monotrauma than in the polytrauma groups (Fig. 3).

### IL-6 and IL-8 transcription in muscle tissues of the “Monotrauma” groups

**Fig. 4:**
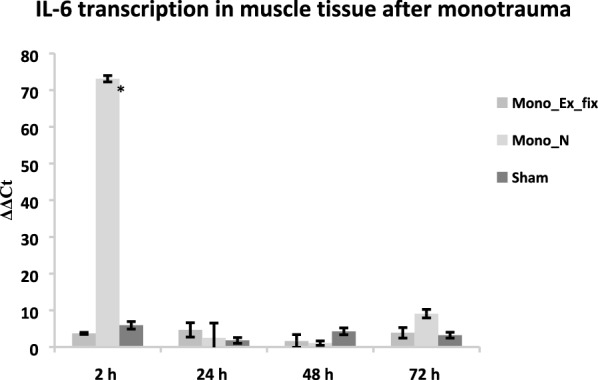
IL-6 transcription in muscle tissue after monotrauma. The qRT-PCR data show significantly increased ΔΔCt values 2 h after trauma induction for monotrauma when using the intramedullary nail compared with the external fixator (*p* < 0.05). No significant transcriptional differences are evident at 24 h, 48 h, or 72 h. Peptidylprolyl isomerase A (PPIA) was chosen as the reference gene

The results for the qRT-PCR are shown as the change in IL-6 transcription represented by the ΔΔCt values. The reference gene for the measurements (housekeeping gene) was the coding gene of peptidylprolyl isomerase A (PPIA) (Fig. 4).

After 2 h, the transcription rates for IL-6 were significantly higher for the Mono_N group (73.13 ± 0.86) than for the Mono_Ex_fix group (3.71 ± 0.28) and the sham group (5.91 ± 1.04). For all other time points and groups measured, there were no significant deviations over the course of 72 h between experimental groups.

**Fig. 5:**
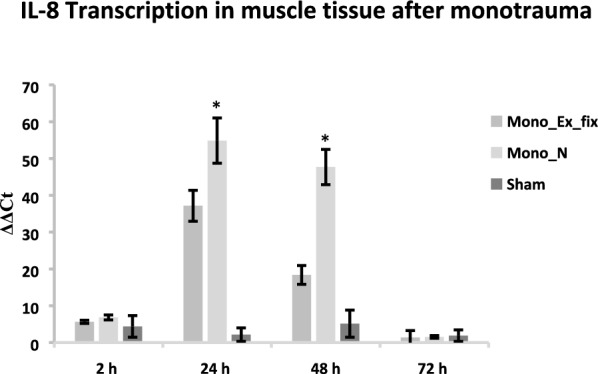
IL-8 transcription in muscle tissue after monotrauma. The transcription levels of IL-8 show significantly increased ΔΔCt values at 24 and 48 h after trauma induction for monotrauma when using the intramedullary nail as compared to the external fixator (*p* < 0.05). At the time points 2 and 72 h, no significant transcriptional differences are evident between the treatment groups and compared to the sham group. Peptidylprolyl isomerase A (PPIA) was chosen as the reference gene

Transcription rates of IL-8 were significantly higher after 24 h and 48 h in group Mono_N (24 h: 54.87 ± 6.14; 48 h: 47.69 ± 4.73) than in group Mono_Ex_fix (24 h: 37.16 ± 4, 48 h: 21; 18.36 ± 2.55). After 48 h, the relative transcriptional increase of IL-8 expression decreased until 72 h. At the beginning and after 72 h, no significant differences in relative transcription between the experimental groups were observed and all values are at the level of the transcription rate measured at the beginning (Fig. 5).

## Discussion

The utmost importance of muscle tissue in fracture healing is well recognized [11]. Muscle tissue provides bones with oxygen, nutrients, and osteoprogenitor cells, but it also seems critical for bone healing due to its immunological potential. In this context, the humeral response in the musculature has been shown to be influenced by trauma severity and by the strategy used for fracture fixation, indicating a potential effect of muscle on the early phase of fracture healing [12]. In the current study, we focused on changes in PMNL infiltration to assess the cellular aspects of the muscular immune response and to elucidate the role of both concomitant injuries and the strategy of fracture fixation in a translationally relevant, long-term pig model. The main results of our study can be summarized as follows:

1. Monotrauma was associated with higher neutrophil counts in the muscle tissue compartment compared with polytrauma, whereas polytrauma resulted in a prolonged PMNL infiltration of the muscle tissue. These relationships were independent of the surgical fracture fixation strategy.
2. Particularly at 24 h after trauma, external fixation was associated with a more pronounced muscular PMNL infiltration pattern than was nailing in monotrauma conditions. In contrast, fracture fixation strategy did not additionally affect the PMNL the migration patterns occurring after polytrauma.
3. Both monotrauma and polytrauma resulted in a delayed increase in PMNL counts in the uninjured muscle of the contralateral femur, with a maximum occurring at 48 h.
4. Nailing after monotrauma resulted in a significant muscular increase in both early IL-6 (2 h after trauma) and later IL-8 (24 h after trauma) transcription.

A well-balanced recruitment of PMNL is of utmost importance for adequate tissue regeneration after trauma. On the one hand, inadequate numbers of migrated PMNL might be associated with an insufficient elimination of pathogens and deficient regenerative processes due to decreased formation of new tissue via neutrophil extracellular trap (NET) structures. On the other hand, excessive PMNL infiltration might damage the surrounding tissue due to the release of reactive-oxygen species (ROS), proteolytic enzymes, and antimicrobial proteins [4, 19]. The increased PMNL infiltration observed here after monotrauma might be explained by a more targeted migration from the systemic circulation into the traumatized tissue in the case of an isolated injury. In contrast, PMNL might spread into different tissues if multiple injuries appear simultaneously. The results from Bastian et al. [7] support this hypothesis, as they found steady decreases in the number of systemic PMNL over the early phase after severe trauma, probably due to a migration into other tissues. They also described an association between a low number of systemic PMNL and delayed fracture healing after multiple trauma, which again underlines the relevance of PMNL in the process of fracture healing [7].

Besides the impact of the trauma severity, our study findings also indicate that the technique used for fracture fixation has a further effect on the extent of muscular PMNL infiltration. However, this association was only found for monotrauma animals and was most pronounced at 24 h. One explanation might be that the stability of fracture fixation influences the local immunological milieu at the fracture site under this condition. In agreement with this assumption, Heiner et al. found an association between flexible stabilization techniques and enhanced gene expression for inflammatory mediators (e.g., IL-6 and heat shock proteins). Specific chemoattractant properties of these mediators might result in an increased PMNL infiltration into muscles [20]. Similarly, Bhatia et al. reported that reamed intramedullary nailing resulted in a more pronounced neutrophil invasion into the systemic circulation compared to external fixation [21]. Systemic recruitment and activation after intramedullary nailing might promote PMNL infiltration in remote organs, as we see trends toward an increase in the AT musculature after monotrauma and nailing. This underlines the effects of undirected PMNL migration, as seen in polytrauma as well.

After polytrauma, external fixation also resulted in a trend toward a higher PMNL count in the musculature, but not before 48 h after trauma. One assumption might be that the effects of concomitant injuries on local and systemic levels of neutrophil granulocytes (e.g., additional infiltration into pulmonary and hepatic tissue) are responsible for these differences between monotrauma and polytrauma. Our findings, and the clinical observation that nailing is clearly the gold standard to assure fracture healing, suggest that the excessive PMNL infiltration into the musculature observed after external fixation is not optimal for the bone healing process. In accordance with this possibility, Simpson et al. found an association between the increased PMNL concentrations within and around the fracture site and the development of non-unions [8]. In contrast, Kovtun et al. found improved fracture healing in cases with higher numbers of PMNL in the fracture hematoma/callus and bronchoalveolar lavage in a multiple trauma (fracture and thoracic trauma) mouse model [9]. Taken together, our findings and those of these previous studies indicate that a precise regulation of PMNL infiltration into different tissues at the fracture site is of extraordinary importance for successful fracture healing and for optimal biomechanical capacity of the bone [22]. A final determination of the influence of the muscular PMNL count on fracture healing will require further studies on a model with a longer posttraumatic observation.

Both traumatic insults (mono- and polytrauma), as well as the methods for fracture fixation (nailing and external fixation), also resulted in an increased PMNL invasion into the musculature of the uninjured extremity (the AT side). When compared with the infiltration in the fracture side, the maximal PMNL infiltration was postponed by 24 h. To the best of our knowledge, this is the first study to describe the infiltration of PMNL into uninjured musculature after an isolated fracture or polytrauma in a translationally relevant large animal project. In accordance with our results, this posttraumatic invasion of PMNL into primarily unaffected tissue has already been shown for different organs, such as the liver and lung [23].

Interestingly, PMNL invasion into the AT-side was not significantly influenced by the trauma severity, as monotrauma animals demonstrated comparable PMNL counts to those experiencing polytrauma. Polytrauma is known to cause a systemic activation of the endothelium, with subsequent invasion of PMNL in different tissues [24, 25], whereas our findings indicate that an isolated femoral fracture and the associated fixation technique are also sufficient for systemic activation of muscle tissues. In agreement, Störmann et al. reported that an isolated fracture resulted in an enhanced PMNL infiltration into the liver and lung. However, in contrast to our findings for the musculature, polytrauma resulted in an intensification of PMNL invasion in those organs, which underlines the high immunological activity of the liver and lungs [26].

Inflammatory mediators and chemoattractants, such as IL-6 and IL-8 are known to play a central role in PMNL activation and migration, respectively. Concomitant injuries have already been reported to affect cytokine transcription at fracture sites [17, 18]; therefore, in the present study, we aimed to independently evaluate the influence of the fracture fixation strategy (nailing vs. external fixation) on the transcription level of IL-6 and IL-8 in the muscles of animals subjected to monotrauma.

When compared with external fixation, intramedullary nailing resulted in a significantly higher IL-6 transcription in the early posttraumatic phase (2 h after trauma); thereby, clearly indicating the greater invasiveness and tissue-damaging effects of this procedure. Our results are in line with those of other studies that showed an increased IL-6 gene expression after intramedullary nailing and other insults (e.g., hyperthermic stress) [27]. In the later stages of our experiment, we observed a rapid decrease in IL-6 transcription. This seems to be of great importance, as persistently high IL-6 concentrations have been associated with impaired bone healing [28, 29], most probably due to an activation of osteoclasts and an associated increase in bone loss [30].

Currently, only very few studies have investigated posttraumatic IL-6 expression in the musculature. Those studies, including those in volunteers after physical activity, described similar courses of IL-6 gene expression to our results [31–34]. When compared with IL-6-transcription, we found the same but delayed association for IL-8, with the highest transcription rates in the nailing group at 24 h after fracture induction and subsequent stabilization. This again reflects the greater tissue damage caused by intramedullary nailing and potentially also the destructive impact on progenitor PMNL cells.

A valid argument could be raised that increased IL-6 and IL-8 transcription in the musculature after femoral nailing would also result in an increased muscular PMNL infiltration compared to external fixation, but this is not the case. The findings of Fielding et al. might provide an explanation, as they described an IL-6-mediated regulation of PMNL trafficking via an activation of STAT3, which in turn downregulates levels of CXCL/KC and could impair PMNL migration into the tissue [35]. Therefore, the increased IL-6 transcription after nailing could also have reduced PMNL infiltration into the musculature in our study. In a further study, Fielding et al. also showed that IL-6 application suppressed IL-1β-induced secretion of IL-8 in an acute peritoneal inflammation model in C57BL/6 J IL-6-deficient (IL-6^−/−^) mice [36]. This could be one of the reasons why the higher IL-8 transcription measured in the Mono_N group is not locally relevant for PMNL infiltration because of the greater inhibition of IL-8 secretion in monotrauma. The higher IL-8 values may not be fully effective due to the concomitantly high IL-6 values.

## Conclusion

Our study is the first to investigate the effects of trauma severity and different fixation treatment strategies of femur fractures on muscular PMNL infiltration in a translationally relevant large animal model. The observed reduction in muscular PMNL infiltration described here after nailing of an isolated femoral fracture suggests that the well-known clinical advantages of intramedullary nailing for fracture healing may be due, at least in part, to the kinetics of PMNL migration into the musculature. After polytrauma, the fixation technique seems to play a minor role in the local recruitment of PMNL.

Nevertheless, the limitation must be mentioned that other cell populations of the immune system such as lymphatic cells, macrophages and mast cells show an equally important and perhaps even opposite mode of action to PMNL near the fracture. To better classify the results presented here, this will be investigated in following large animal projects.

## Data Availability

Data sharing is not applicable to this article as no datasets were generated or analyzed during the current study.
